# Structural and functional characterization of a highly stable endo-β-1,4-xylanase from *Fusarium oxysporum* and its development as an efficient immobilized biocatalyst

**DOI:** 10.1186/s13068-016-0605-z

**Published:** 2016-09-05

**Authors:** Sara Gómez, Asia M. Payne, Martin Savko, Gavin C. Fox, William E. Shepard, Francisco J. Fernandez, M. Cristina Vega

**Affiliations:** 1 Structural and Quantitative Biology Department, Center for Biological Research (CIB-CSIC), Ramiro de Maeztu 9, 28040 Madrid, Spain; 2 Synchrotron SOLEIL, L’Orme des Merisieris Saint-Aubin BP48, 91192 Gif-sur-Yvette, France; 3 Department of Immunology, Complutense University School of Medicine, Madrid, Spain; 4 Abvance Biotech srl, Madrid, Spain

**Keywords:** Bioethanol, Docking, Family 11 glycoside hydrolase (GH11), Immobilization, Lignocellulosic biomass, Structural biology, X-ray crystallography, Xylanase

## Abstract

**Background:**

Replacing fossil fuel with renewable sources such as lignocellulosic biomass is currently a promising alternative for obtaining biofuel and for fighting against the consequences of climate change. However, the recalcitrant structure of lignocellulosic biomass residues constitutes a major limitation for its widespread use in industry. The efficient hydrolysis of lignocellulosic materials requires the complementary action of multiple enzymes including xylanases and β-xylosidases, which are responsible for cleaving exo- and endoxylan linkages, that release oligocarbohydrates that can be further processed by other enzymes.

**Results:**

We have identified the endo-β-1,4-xylanase Xyl2 from *Fusarium oxysporum* as a promising glycoside hydrolase family 11 enzyme for the industrial degradation of xylan. To characterize Xyl2, we have cloned the synthetic optimized gene and expressed and purified recombinant Xyl2 to homogeneity, finally obtaining 10 mg pure Xyl2 per liter of culture. The crystal structure of Xyl2 at 1.56 Å resolution and the structure of a methyl-xylopyranoside Xyl2 complex at 2.84 Å resolution cast a highly detailed view of the active site of the enzyme, revealing the molecular basis for the high catalytic efficiency of Xyl2. The kinetic analysis of Xyl2 demonstrates high xylanase activity and non-negligible β-xylosidase activity under a variety of experimental conditions including alkaline pH and elevated temperature. Immobilizing Xyl2 on a variety of solid supports enhances the enzymatic properties that render Xyl2 a promising industrial biocatalyst, which, together with the detailed structural data, may establish Xyl2 as a platform for future developments of industrially relevant xylanases.

**Conclusions:**

*F. oxysporum* Xyl2 is a GH11 xylanase which is highly active in free form and immobilized onto a variety of solid supports in a wide pH range. Furthermore, immobilization of Xyl2 on certain supports significantly increases its thermal stability. A mechanistic rationale for Xyl2's remarkable catalytic efficiency at alkaline pH is proposed on the basis of two crystallographic structures. Together, these properties render Xyl2 an attractive biocatalyst for the sustainable industrial degradation of xylan.

**Electronic supplementary material:**

The online version of this article (doi:10.1186/s13068-016-0605-z) contains supplementary material, which is available to authorized users.

## Background

The raising global demand for renewable fuels, commodity and platform chemicals and polymeric materials, together with the global climate change so strongly associated with the consumption of fossil fuels, are considered in all scientific and political agendas as some of the biggest societal challenges for the next decades [1, 2]. Nevertheless, research and industry efforts to find new energy resources that can substitute fossil fuel have thus far been curbed by sustainability limitations identified for the first-generation biofuels. These limitations have promoted the development of second-generation biofuels based on the use of wood, grass, agricultural and forest lignocellulosic residues and fiber sludge as resources for obtaining bioethanol [3]. The enormous potential shown by this second wave of biofuels suggests that biomass could become a key resource for renewable energy supply based on future biorefineries [3–6].

**Fig. 1:**
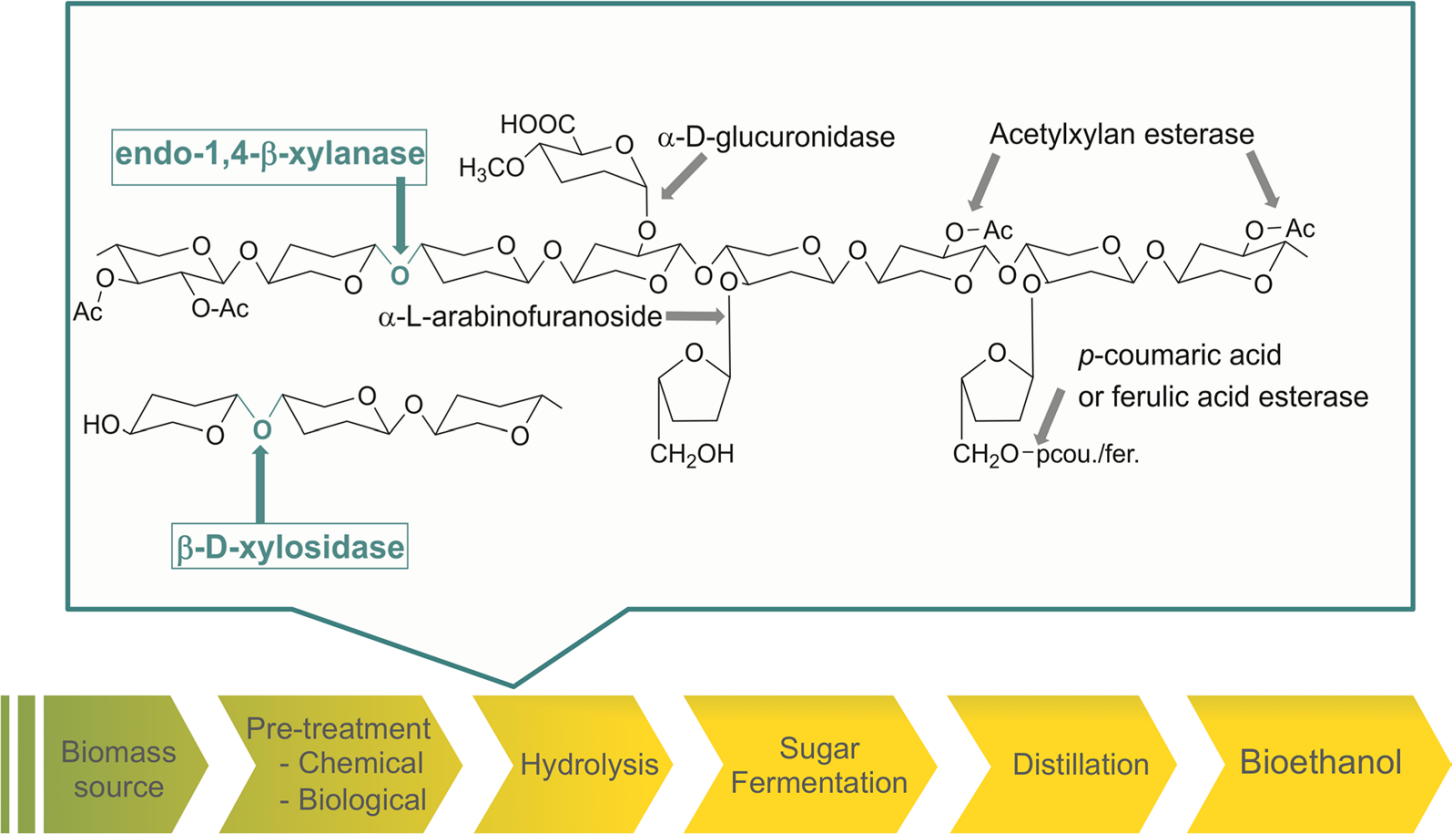
Schematic representation of bioethanol production using lignocellulosic biomass residues. *Bottom*, flowchart representing the main steps in the chemical/physical and enzymatic production of bioethanol. *Top*, close-up of the multi-enzymatic hydrolysis step, showing the xylan structure as composed mainly of 1,4-β-linked xylose residues and the various enzymes implicated in its degradation. *Ac* acetyl group; *p*cou *p*-coumaric acid; *fer* ferulic acid

Use of lignocellulosic biomass as biofuel source is based on a two-step enzymatic technology, whereby complete biomass biodegradation requires multiple collaborative enzymes (Fig. 1) [7–9]. Current limitations in the broad application of lignocellulosic biomass for biofuel production stem from the intricate and complex plant cell-wall structure [10, 11]. Lignocellulose is a major component of plant cell-wall architecture, consisting of cellulose, hemicellulose and lignin [12]. Hemicellulose, the second most abundant constituent, is mainly composed of xylan, a linear backbone polymer of β-d-xylopyranosyl units linked by β-(1,4) glycosidic bonds, commonly branched with 4-*O*-methyl-α-d-glucuronopyranosyl units, acetyl groups or α-l-arabinofuranosyl units, in variable proportions [3–6]. Complete depolymerization of xylan requires the joint action of different enzymatic activities, including endo-1,4-β-xylanases (EC 3.2.1.8), β-d-xylosidases (EC 3.2.1.37), α-l-arabinofuranosidases (EC 3.2.1.55), α-glucuronidases (EC 3.2.1.139), acetyl xylan esterases (EC 3.1.1.72), and ferulic/coumaric acid esterases (EC 3.1.1.73) [13]. Among them, endo-1,4-β-xylanases play a crucial role for their implication in breaking down the xylan backbone in smaller, less recalcitrant fragments, thereby increasing the overall rate of release of fermentable sugars and making cellulose more accessible for further hydrolysis or fermentation [6, 11, 14]. The complex branching and acetylation patterns so characteristic of plant cell-wall structures give lignocellulosic biomass a recalcitrant nature, which is the main reason responsible for the high cost associated with lignocellulosic conversion [11, 15]. After chemical or physical pretreatment to reduce lignin, the lignocellulosic cell-wall polysaccharide has to be deconstructed by enzymatic hydrolysis processes that liberate fermentable carbohydrates to the medium. At this stage, downstream enzymes can ferment those sugars to produce bioethanol and residual biomass, which can be further purified by distillation [15]. A more sustainable and cost-efficient pretreatment of lignocellulosic biomass that is under intense scrutiny employs ionic liquids (ILs) such as 1-ethyl-3-methylimidazolium acetate ([Emim]OAc) to dissolve it [16]. The dissolution in ILs is supposed to initiate structural changes in the regenerated biomass by lowering the cellulose crystallinity and lignin content, resulting in its deconstruction to facilitate the extraction of biomass carbohydrates for their utilization [17, 18].

Fungal plant pathogens are considered promising sources of cell wall-degrading enzymes [19]. The *Fusarium oxysporum* is well known as a major crop plant-pathogenic ascomycete whose genome encodes a complete xylanolytic degradative system, an arsenal of cell wall-degrading enzymes (CWDE) that allows it to efficiently convert plant biomass (cellulose and xylan) into ethanol [20, 21]. At least six different β-(1,4)-xylanases have been discovered in the *F. oxysporum* genome belonging to the GH10 and GH11 families [22–27], which have been characterized and discussed in the context of their role in pathogenesis. Their high activity and overall efficiency at degrading plant cell-wall components make *F. oxysporum* xylanase a repertoire of strategic potential for the biofuel industries [21]. Nevertheless, the native form of xylanases does not usually meet the strict industrial requirements for large-scale biofuel production. Hence, directed evolution methods have been applied to naturally occurring xylanases to isolate mutants with increased thermal and pH resistance and for the selection of overexpressing strains. In parallel, immobilization on multiple supports has been applied to xylanases from multiple microbial and fungal sources as an indirect way to enhance the thermal and pH stability of natural and mutant xylanases [28–30]. In particular, in *Fusarium,* many efforts have been made for culture and activity optimization to find alternative approaches that reduce cost limitations [31–35].

Here, we describe the crystal structure and enzymatic properties of Xyl2, a novel GH11 endo-β-1,4-xylanase from the pathogenic fungus *F. oxysporum* that also exhibits a synergic β-d-xylosidase activity. Xyl2 has been further developed into an efficient, alkaline pH-tolerant, thermostable immobilized biocatalyst which shows promise for sustainable industrial bioprocesses around the enzymatic degradation of xylan-containing biomass sources.

## Results and discussion

### Phylogenetics

**Fig. 2:**
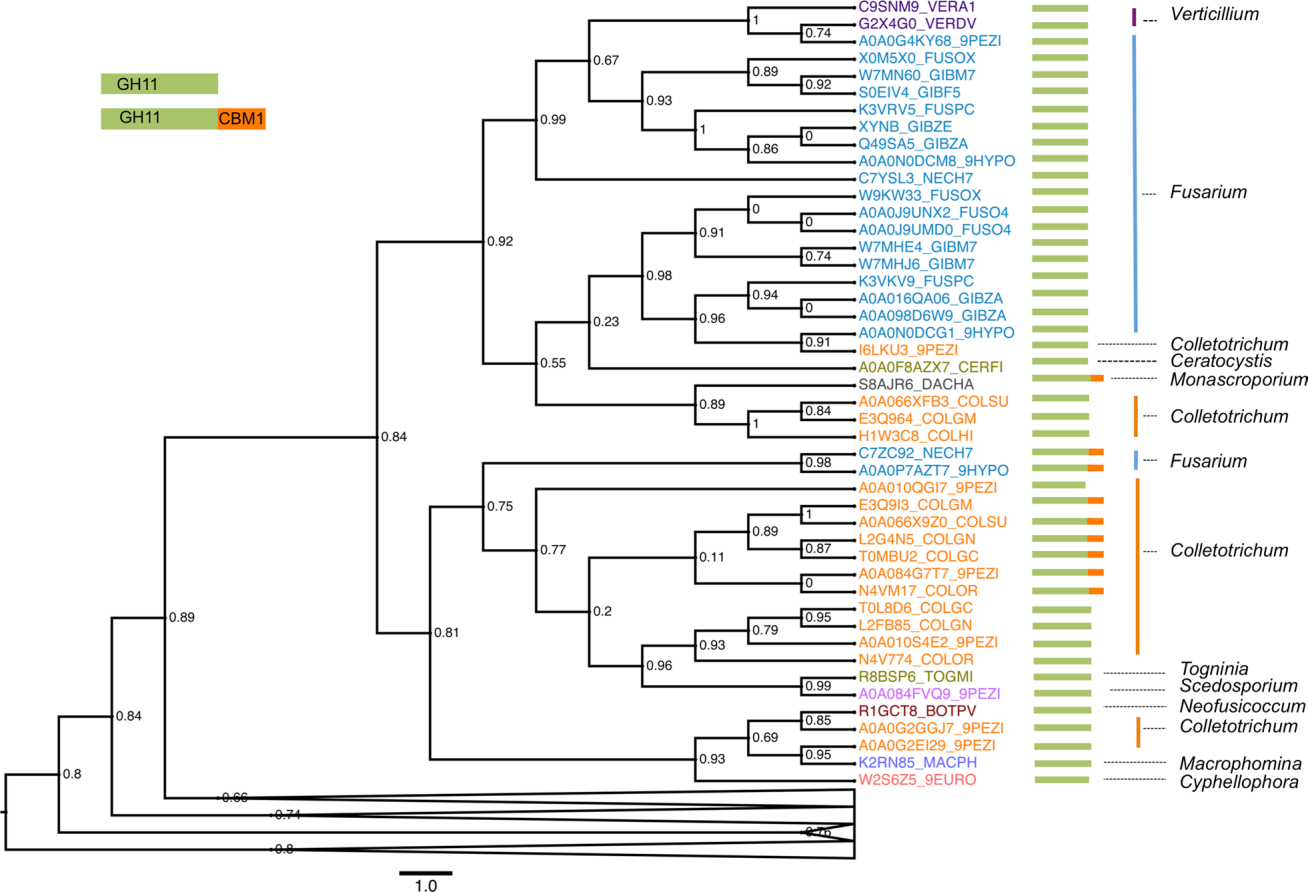
Phylogenetic tree of fungal xylanases sequences. Unrooted phylogenetic tree of selected xylanase sequences calculated with the maximum-likelihood software PhyML and depicted with FigTree with branch supports shown at every node. The UniProt code names of Xyl2 homologs present in the genomes of *Fusarium* spp. are shown in *blue*, with those of more distantly related homologs colored differently. The domain architecture of each GH11 sequence shown is schematized next to the enzyme name with a *green rectangle* for the GH11 domain fold and the CBM1 domain in *orange*. Folded branches include additional related sequences that exhibit less evolutionary relatedness

Sequences encoding glycoside hydrolase family 11 (GH11) endoxylanases, including sequences of known enzyme structure, were retrieved with the BLASTp [36] software using *F. oxysporum* endo-β-1,4-xylanase Xyl2 (UniProt entry X0M5X0_FUSOX) as query against a non-redundant database of protein sequences. The hit sequences (250 sequences) were then filtered to remove identical pairs and the N-terminal peptide leader sequence was trimmed. The remaining sequence pool containing 163 unique sequences were aligned with T-Coffee [37] using default parameters, and the aligned sequences were analyzed with the maximum-likelihood PhyML [38] software to reconstruct their phylogenetic relations (Fig. 2). Phylogenetic trees were displayed and annotated with FigTree v1.4 [39].

The sequence alignment and phylogenetic analyses of the homologous sequences showed that the fungal Xyl2 sequences available shared significant sequence similarity (often above 60 %) and that they cluster tightly around a variety of related *Fusarium* spp. (commonly known as *Gibberella*, *Nectria* and *Hypocrea*) and *Colletotrichum* spp., and a few other fungal species belonging to *Verticillum* and the more distantly related *Togninia*, *Scedosporium*, *Neofusicoccu*, *Acrophomina* and *Cyphellophora* (Fig. 2). In addition to the shared GH11 core domain, some endo-1,4-β-xylanases from *Fusarium* (*Nectria* and *Hypocrea*) present the C-terminal fungal-specific carbohydrate-binding module (CBM1 domain) (Fig. 2). This domain is also present in other xylanases from *Colletotrichum* and *Monacrosporium* spp. The fungal CBM1 domain is of great interest in biotechnology since it functions to position the carbohydrate-active domain in close proximity to the xylan substrate, therefore increasing the catalytic efficiency of the enzyme.

The remarkable degree of conservation of gene length and catalytic amino acid residues all argue in favor of the existence of a common ancestral enzyme sequence to the GH11 family of enzymes. This evolutionary landscape is further expanded by the appearance by divergence evolution in many fungal species of the additional C-terminal CBM1 domain. Together, these observations highlight the relevance of xylan-degrading enzymes across fungi.

### Cloning, expression and purification

**Fig. 3:**
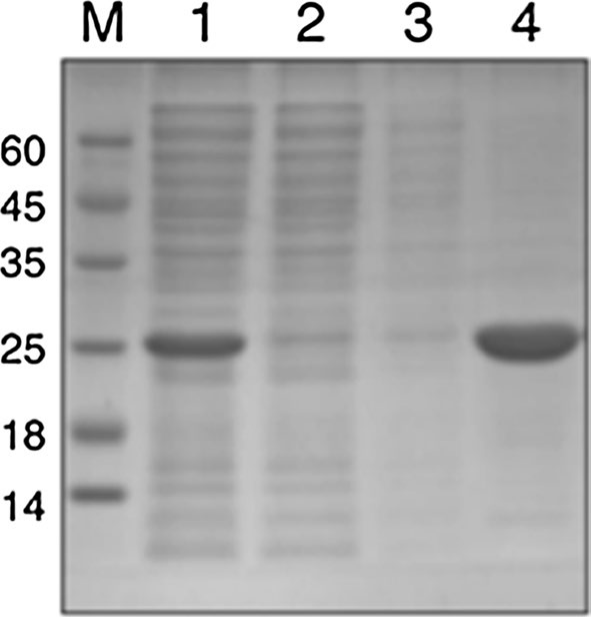
Purification of Xyl2. Coomassie brilliant blue (CBB)-stained 15 % SDS-PAGE gel electrophoresis of the purification of Xyl2 by nickel affinity chromatography. The gel was stained with coomassie brilliant blue R-250. *M* molecular size marker. *Lane 1* crude supernatant applied to the affinity resin, *Lane 2* flow-through, *Lane 3* wash, *Lane 4* elution of Xyl2

The DNA sequence encoding full-length *F. oxysporum* endo-β-1,4-xylanase Xyl2 (EC 3.2.1.8), except for the peptide leader (amino acid residues 1-19), spanning residues 20-232, was cloned in the pETM-11 expression plasmid and transformed into *Escherichia coli* BL21(DE3) for recombinant expression. A highly pure and active enzyme preparation was obtained after harvesting the cell pellet from expression cultures and purifying Xyl2 from the soluble lysate fraction by one-step nickel-affinity chromatography. The N-terminal tobacco etch virus (TEV)-cleavable hexahistidine tag was optionally removed by overnight digestion with rTEV (1:10 mass ratio) followed by a second subtractive step with nickel-affinity resin where untagged Xyl2 was collected in the flow-through. The purity and the molecular weight of Xyl2 prior to (24.2 kDa) and after the proteolytic removal of the hexahistidine tag (21.2 kDa) were confirmed by coomassie brilliant blue (CBB)-stained SDS-PAGE gel electrophoresis (Fig. 3).

### Overall crystal structure of unliganded Xyl2

**Fig. 4:**
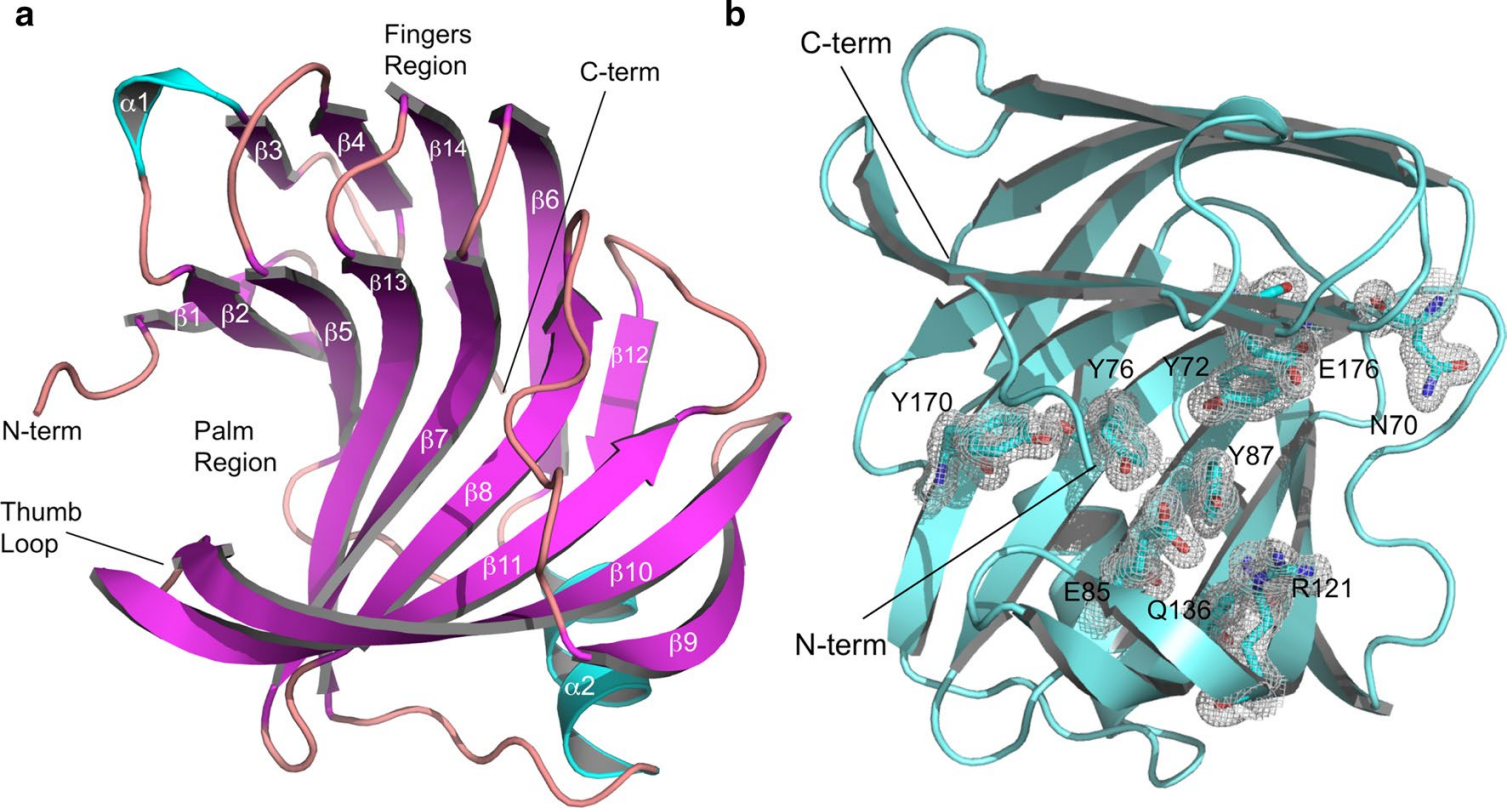
Overall structure of unliganded *F. oxysporum* Xyl2. **a** Ribbon representation of the unliganded Xyl2 crystal structure at 1.56 Å resolution with annotated secondary structure elements. The N- and C-termini are labeled. Helix 3_10_A and α1 are colored in cyan, β-strands in *pink*, and interconnecting loops and the extremes are shown in *salmon*. The orientation shown provides a view into the wide and elongated active-site *groove*, which was filled with water and solvent molecules in the crystal structure. **b** Overall ribbon structure of Xyl2 (in *cyan*) showing residues thought to interact with the cognate xylan substrate are shown as sticks in the electron density map (contoured at 1σ level). Compared with the orientation shown in **a**, this orientation is slightly rotated clockwise around a *vertical axis*

The crystallographic structure of Xyl2 at 1.56 Å was determined by molecular replacement and refined to convergence with *R*/*R*_free_ values of 0.186/0.234 (Table 1). The final model contains the complete amino acid sequence except for the first and last amino acids (residues 2-190). Overall, Xyl2 folds into the characteristic GH11 xylanase fold consisting of a highly twisted β-sandwich core that comprises 14 β-strands and is further stabilized by a single α-helix in the opposite side of the active site (Fig. 4a; Additional file 1: Figure 1). The groove delimited by the long β-hairpin, commonly denominated the “thumb” region, and the curved side of the β-sandwich or “fingers” region defines a long, wide valley into which the xylan substrate molecule binds, or “palm” region (Fig. 4a). The residues at the base of the groove constitute the palm region. Up to six xylose units have been shown to fit into the active site of homologous xylanases although the xylose units located in the ends are poorly bounded [40, 41]. The N-terminus of Xyl2, however, adopts a particular conformation and, instead of running away from the active site, it folds into an elongated motif that forms one side of the active-site groove (Fig. 4). Since the position and orientation of this unique feature appear to complete the fingers in Xyl2, we propose to call this unique feature the “pinky” motif. The configuration of the residues in the pinky motif (residues 2–6) is well defined in electron density maps, thereby representing a stable conformation for the Xyl2 N-terminus.

**Table 1:** Crystallographic data collection and refinement statistics

PDB code	*Fo*Xyl2	*Fo*Xyl2/MBX
	5JRM	5JRN
Synchrotron source	SOLEIL/PX 2A	ALBA/XALOC
*Data collection*		
Wavelength	0.9801	0.9801
Resolution range (Å)	29.65–1.56 (1.616–1.56)	48.75–2.84 (2.94–2.84)
Space group	*P* 21 21 2	*P* 21 21 2
Unit cell (Å,º)	105.2, 35.9, 48.3, 90, 90, 90	105.7, 36.1, 48.7, 90, 90,90
Total reflections	187,439 (17,857)	44,904 (4368)
Unique reflections	26,596 (2559)	4766 (450)
Multiplicity	7.0 (7.1)	9.1 (8.7)
Completeness (%)	99 (97)	100 (100)
Mean *I*/σ(*I*)	13.91 (1.89)	12.96 (3.73)
Wilson *B*-factor	16.52	35.89
R-merge	0.099 (0.901)	0.252 (0.884)
R-means^a^	0.108 (0.972)	0.266 (0.930)
CC_1/2_^b^	0.998 (0.607)	0.989 (0.706)
CC*^c^	0.999 (0.869)	0.997 (0.930)
*Refinement*		
Reflections used in refinement	26,593 (2559)	4760 (449)
Reflections used for R_free_	1338 (115)	239 (23)
R_work_	0.186 (0.340)	0.209 (0.329)
R_free_	0.234 (0.345)	0.227 (0.441)
CC_work_	0.968 (0.609)	0.938 (0.725)
CC_free_	0.941 (0.707)	0.922 (0.660)
Number of non-H atoms	1722	1547
Macromolecules	1535	1473
Ligands	21	53
Protein residues	189	189
RMS(bonds) (Å)	0.007	0.005
RMS(angles) (º)	1.12	0.93
Ramachandran favored (%)	95.5	95.2
Ramachandran allowed (%)	4.0	4.8
Ramachandran outliers (%)	0.5	0.5
Rotamer outliers (%)	0.6	0.0
Clashscore	2.00	7.48
Average *B*-factor (Å^2^)	24.35	38.35
Macromolecules	22.52	37.19
Ligands	84.39	72.53
Solvent	33.65	33.40

^a^R-means = Σ_hkl_ (n/n−1)^1/2^ Σ_i_|I_i_(hkl)−<I(hkl)>|/ΣΣ_i_ I_i_(hkl); where i is the ith measurement of reflection (hkl) and < I(hkl) > is the average over symmetry-related observations of a unique reflection (hkl)

^b^CC_1/2_ is the Pearson correlation coefficient calculated between two random half data sets

^c^CC* is the CC of the full data set against the true intensities, estimated from CC* = [2 CC_1/2_/(1 + CC_1/2_)]^1/2^

As expected, the critical active site residues of GH11 xylanases are conserved in Xyl2. The general mechanism of glycoside hydrolases (GH) starts with the nucleophilic attack by a carboxylate nucleophile (Glu176 side chain) on the anomeric carbon of the scissile glycosidic bond, resulting in the release of the linked sugar residue and the formation of a covalent enzyme–sugar intermediate. In a second step, an activated water molecule displaces the side chain of a second glutamate residue that acts as a general acid/base catalyst (Glu85) in a process that retains the original stereochemical configuration at the anomeric carbon. The hydroxyl function of neighboring tyrosine (Tyr72 and Tyr76) side chains interact with the xylan chain and contribute to the correct positioning and orientation of the substrate. Besides Tyr76, the residues in the active site from the palm region (Tyr179, Gln136, Arg121 and Asn70) are fully conserved across GH11 xylanases and entirely defined in the electron density map at 1.56 Å contoured at 1σ level (Fig. 4b).

### Crystal structure of Xyl2/MBX

**Fig. 5:**
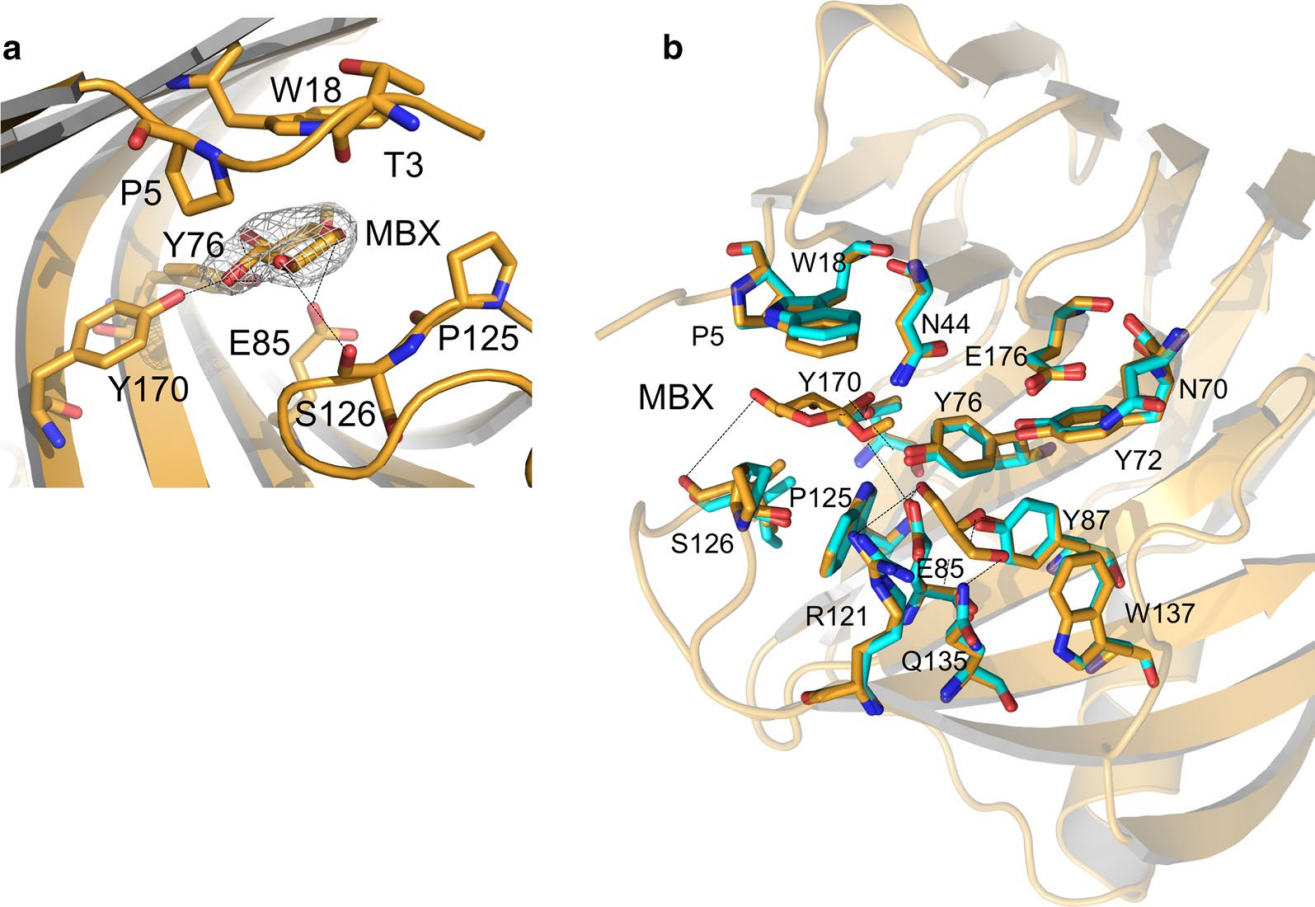
Structure of *F. oxysporum* Xyl2 in complex with methyl-β-xylopyranoside (MBX). Ribbon representation of Xyl2 (in *gold*) in complex with MBX. **a** Close-up of the MBX binding pocket, highlighting the role of Xyl2 N-terminus (the “pinky” motif at the margin of the fingers region, spanning Thr3-Gln4-Pro5) in stabilizing the bound conformation of MBX. The MBX molecule is represented as sticks and overlaid with the electron density map (contoured at 1σ). Important hydrogen bond interactions are shown as *dashed lines*. **b** The substrate-binding residues are shown in sticks for the MBX complex structure (*gold*) and also for the unliganded structure (in cyan), for comparison. Hydrogen interactions are shown as *dotted lines*

To shed light into the catalytic mechanism of Xyl2, we determined its crystal structure with methyl-β-d-xylopyranose (MBX) at 2.84 Å resolution from Xyl2 crystals soaked with a concentrated solution of the monosaccharide for 5 min. After soaking with MBX, the crystals were immersed in a drop of crystallization condition with 10 % of glycerol added as cryoprotectant and frozen in liquid nitrogen. The Xyl2/MBX crystal structure was solved by molecular replacement using the refined structure of unliganded Xyl2 as model. Upon convergence, the *R*/*R*_free_ values were 0.209/0.227 (Table 1), and the refined Xyl2/MBX model contains residues 3–190. Omit maps calculated with molecular replacement phases before refinement revealed the presence of additional electron density in the active site pocket consistent with the presence of one MBX unit bound to Xyl2 (Fig. 5a). The precise location of MBX coincides with the position-2 that would have been occupied by a longer xylan chain, as seen in the crystal structure of Xyn11 from *T. reesei* (*Tr*Xyn11) in complex with a hexasaccharide substrate (PDB code 4hk8) [40]. Indeed, in the latter structure, the xylose unit at position-2 makes a significantly greater number of sugar-enzyme interactions than the remaining xylose units. Hence, Xyl2 residues within interaction distance to MBX might act as anchoring points for stable xylan binding, thereby serving a guiding or orientating purpose. The hydrogen bonding interactions between MBX and Glu85, Ser126, Tyr170 and Tyr176 are all located in a cavity encircled by the side chains of Pro5 (from the pinky motif) and the side chains of Trp18, Asn44 and Pro125 (Fig. 5a, b). The entry of MBX in the active site is followed by some small adaptations like the displacement by 0.65 Å of Pro125 and Ser126 in the thumb region and the rotation of about 10º in the relative orientation of the indole ring of Trp18 side chain, which was also observed in the structure of *Tr*Xyn11 in complex with the hexaxylose ligand (Fig. 5b).

Comparison of the Xyl2/MBX and the unliganded Xyl2 active-site structures revealed that, in the unliganded Xyl2, a glycerol molecule from the cryoprotectant solution was found in the active site near to the position occupied by MBX ligand (Fig. 5). The glycerol molecule is stabilized by interactions with the side chains of the catalytic residues Tyr87, Arg121 and Gln135, and is well defined in the electron density map, which reinforces the view that Xyl2 active site is particularly capable of binding xylose and chemically related molecules situated at position-2, the position of the xylene subunit immediately adjacent to the β-(1,4) glycosidic bond to be hydrolyzed (Fig. 5). The conserved binding xylan sequence motif (Pro–Ser–Ile) located at the tip of the thumb-loop plays a key role in substrate specificity, being involved in the interactions of −2 and −1 xylosyl units. Tyr122 and Thr133 control the movement of the thumb loop; therefore the substitution of Tyr122 by smaller residues (e.g., Ser or Thr) facilitates the flexibility of the loop and, in consequence, the turnover of the substrate.

### Structural homology

**Fig. 6:**
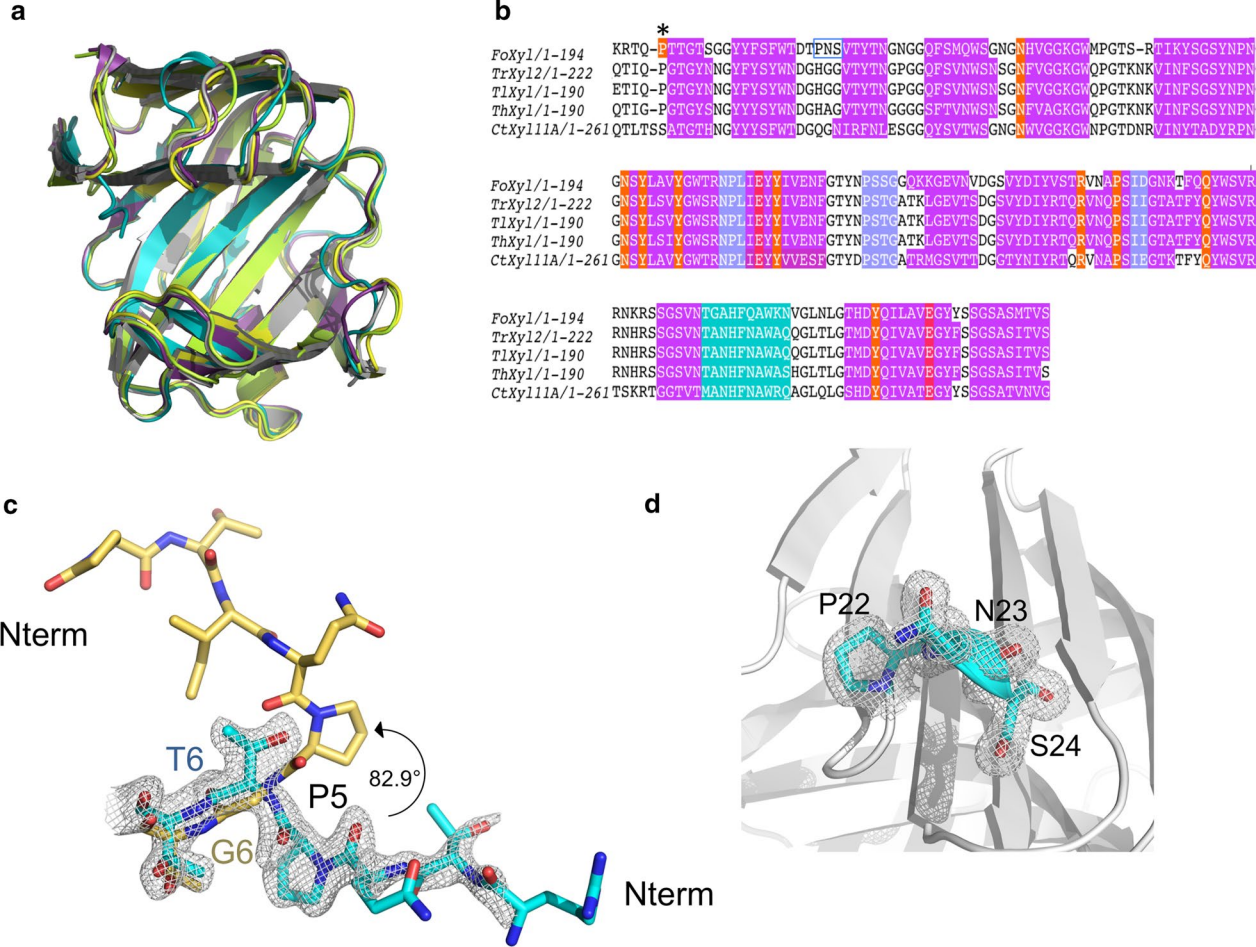
Structural homology of *F. oxysporum* Xyl2 with fungal GH11 xylanases. **a** Superposition of *F. oxysporum* Xyl2 with structural homologous GH11 xylanases of fungal origin, including *Trichoderma reesei* Xyl2 (*Tr*Xyl2, *yellow*), *T. longibrachiatum* Xyl2 (*Tl*Xyl2, *violet*), *T. harzianum* Xyl2 (*Th*Xyl2, *gray*) and *Chaetomium thermophilum* Xyl11A (*Ct*Xyl11A, *green*). **b** Multiple sequence alignment with secondary structural elements overlaid in *pink* (β-strands) or cyan (helix α1), and the 3_10_A helix motif (Pro22-Asn23-Ser24) is* boxed* with a* blue* outline. An* orange* background represents active site residues. An* asterisk* is shown on top of Pro5 to indicate that it participates in the substrate binding thanks to the unusual conformation of the N-terminal end of Xyl2. **c** Comparison of the atypical conformation adopted by the N-terminal extreme of Xyl2 (in *cyan*) in comparison with all other GH11 xylanases (in *yellow*), with the electron density map (contoured at 1σ) overlaid to support the observed configuration. **d** Close-up onto the 3_10_A helix motif showing the electron density map (contoured at 1σ), connecting β-strands β2 and β3 by a tight turn

*F. oxysporum* Xyl2 is structurally and sequence homologous with Xyn11 from *Trichoderma* spp. (*T. reesei*, *T. harzianum* and *T. longibrachiatum*) with r.m.s. distance values after superposition of 183 Cα atoms (residues 6–188) of 0.71, 0.74 and 0.76 Å, respectively (Fig. 6a, b). Structural and sequence similarities are also noticeable between Xyl2 and the homologous xylanase from *Colletotrichum* sp. with an r.m.s. distance of 0.77 Å (184 Cα atoms between 6–189) (Fig. 6a, b). The fold is unequivocally conserved, including the overall fold plan, the *β*-turns between β7–β8, β8–β9 and β11–β12, and the ten-amino-acid residue α-helix opposite to the palm region (Figs. 4, 6a).

However, there are two unique features in the crystal structure of Xyl2 that are lacking in the GH11 xylanase structures known so far. First, the conformation of the N-terminus is very distinctive (Fig. 6c). In the closest homologs (*Trichoderma* and *Colletotrichum* spp. Xyn11), the presence of a Gly at position 6 facilitates the rotation of the backbone by up to approximately 83° with respect to the N-terminal end. In contrast, in Xyl2, a Gly-to-Thr substitution at position 6 stabilizes a straighter path for the backbone direction, pushing it downwards and to the flank of the active-site groove. In this location, the N-terminus could establish further interactions with the substrate to restrict or guide its bound conformation. Indeed, the MBX ligand in the Xyl2/MBX structure can interact with the N-terminal residue Pro5, an interaction unseen in the homologous structures. Furthermore, Thr3 is only within 4 Å from MBX, suggesting that additional interactions with the N-terminus will be possible with longer substrates harboring xylose units at position-3 of the xylan chain. Second, it is also noticeable the existence of the unique 3_10_ helix (Fig. 6d; also shown labeled α1 in Fig. 4a and boxed in Fig. 5b), which comprises a PNS sequence motif (Pro22-Asn23-Ser24) between strands β2 and β3. The 3_10_ helical residues are well defined in the electron density in both the unliganded and MBX complex structures (Fig. 6d).

**Fig. 7:**
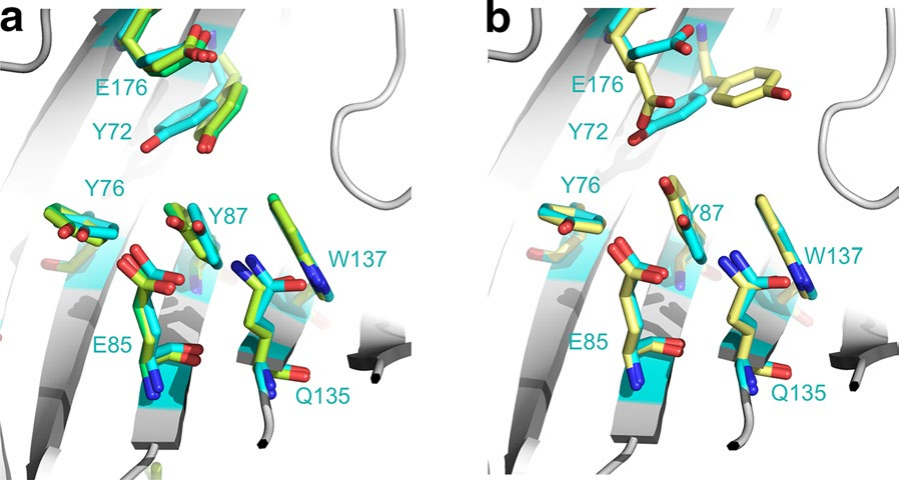
Active site configuration at acidic and alkaline pH. Comparison of the active site configuration at two extreme pH conditions by superposing the crystal structures of *F. oxysporum* Xyl2 obtained at pH 5.0 (*cyan*) with the neutron crystal structures of *T. reesei* Xyl2 at pD 4 (*olive green*), pD 4.4 (*light green*) **a** or pD 8 (*pale yellow*) **b** The rotameric conformations of Tyr73 and Glu177 (numbering according to *T. reesei* Xyl2) are flipped over at the two extreme pH values, with catalytically competent conformations being observed only at alkaline pH conditions (**b**)

It is interesting to note that in the structure of Xyl2, which was determined from high-quality crystals grown at pH 5, the rotameric conformations adopted by Tyr72 and Glu176 side chains correspond to a catalytically less efficient configuration (Fig. 7a). In the observed conformations, Glu176 side chain points to the bulk solvent, which causes the side chain of Tyr72 to concertedly flip over to a less hindered conformation (Fig. 7a,b). This particular conformation for Glu176/Tyr72 has been observed before in the neutron crystal structure of *T. reesei* Xyn11 at pD4–4.4 with an empty active site (PDB 4s2f), which was shown to be able to return to a catalytically competent conformation at basic *pD* values [40, 42].

### Docking

**Fig. 8:**
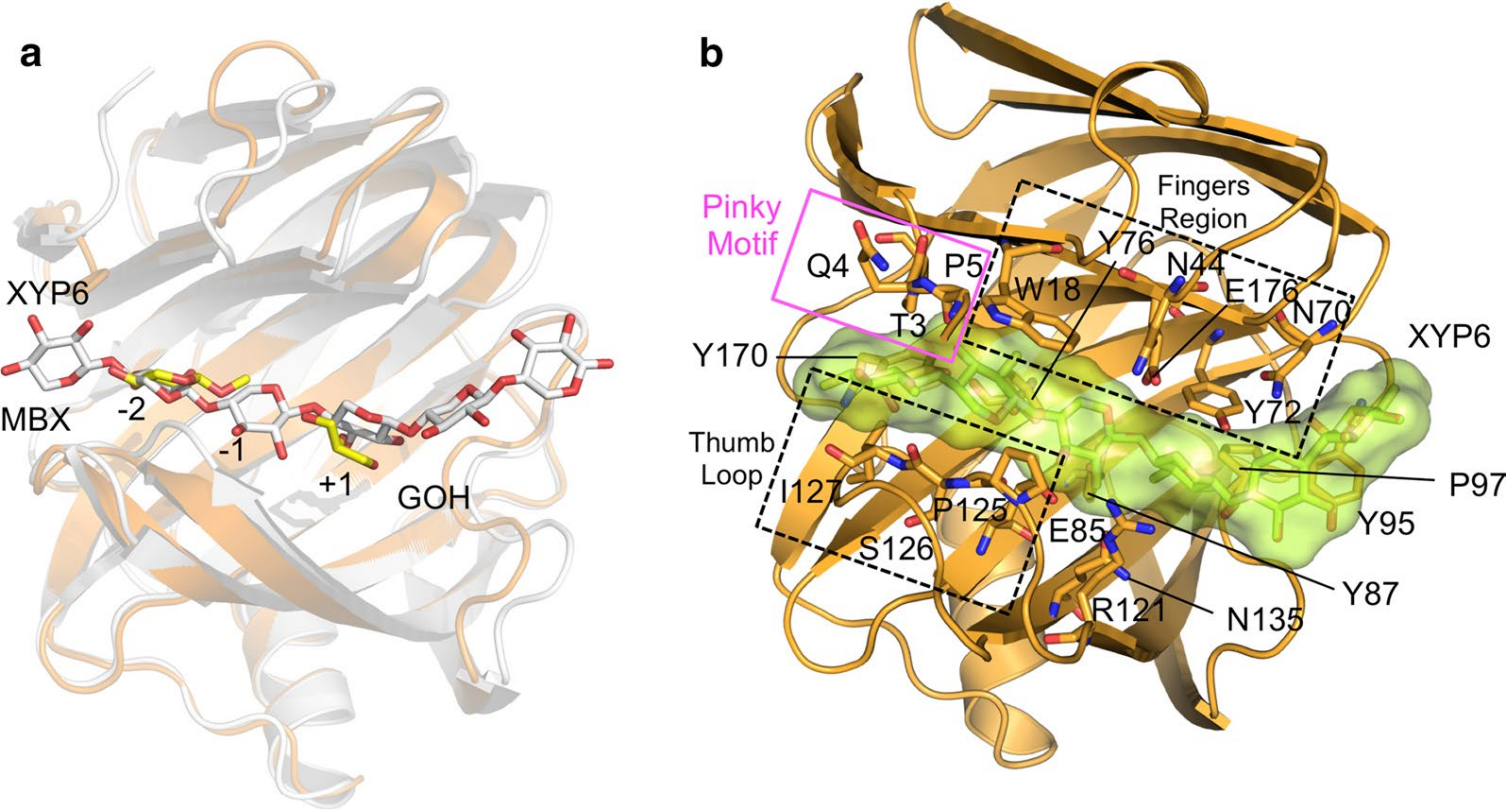
Modeled Xyl2/hexaxylose oligosaccharide complexes. Modeled Xyl2 substrate complexes were built from **a** the direct superposition of Xyl2/MBX and *Tr*Xyn11 in complex with a hexaxylose oligosaccharide (PDB 4hk8) and **b** by systematic docking experiments starting with the initial conformation shown in (**a**). The structure of Xyl2 is shown in cartoon representation (*orange*), with catalytic and ligand-binding substrates shown in *sticks* and *labeled*. The bound ligand is represented in sticks (C atoms are *white*) and as a semi-transparent molecular surface (in *lemon color*)

To further our understanding of Xyl2 substrate binding and catalytic mechanism, we used the Xyl2/MBX crystal structure as a starting point for docking experiments conducted with AutoDock Vina [43, 44]. In control docking runs with MBX, we had previously verified that the binding pose of least free energy was coincident with the observed pose of MBX in the crystal structure. In that pose, the orientation of MBX is very close to that observed for sugar units of longer substrates at the same position, thus indicating that the MBX binding pose was biologically relevant. To initiate the docking runs with a hexaxylose molecule, we superimposed a relevant ligand complex (PDB 4hk8) [40] with Xyl2 (r.m.s. distance 0.82 Å for 184 Cα atoms), isolated the hexasaccharide ligand conformation and merged it into the Xyl2 structure (Fig. 8a). In the present crystal structure, the MBX ligand and a glycerol molecule from the cryoprotectant are occupying two subpockets (−2 and +1, respectively) along the hexasaccharide-binding groove. The first or head subpocket, located in the deepest section of the active site, targets the xylopyranoside unit at position-2. The −1 section or core site, which is more extensive, is responsible for binding the central xylan units and performing the catalytic cleavage. In addition, a similar search with the crystallographic *Tr*Xyn11/hexaxylose model structure yielded a least free-energy pose that was essentially identical to the true binding pose, therefore proving the reliability of the docking protocol. The resultant hybrid model was used as the starting point for subsequent docking runs. During docking runs, we simulated pH 5 (where the enzyme is poorly active) and pH 7 (in which the enzyme is active). The present structure confirms the poor activity for Xyl2 at this pH since it presents a concerted change in the Glu176 and Tyr72 rotamers, with the nucleophilic Glu176 side chain being kept away from the substrate (Additional file 1: Figure S2). At pH 7.5, where the enzyme is active, both residues revert to a catalytically effective conformation (Figs. 7b, 8a).

From the resulting docking poses of the hexasaccharide into the Xyl2 active site (Fig. 8b), the lowest energy poses of the hexasaccharide ligand result in conformations that are compatible with the hydrolysis of the *β*-1,4-glycosidic bond between units −1 and +1, while the sugar unit placed at position-2 has the greatest buried surface. Furthermore, the docked poses make strong hydrogen bond interactions with the nucleophilic and acid/base glutamate residues Glu176 and Glu85, respectively (Fig. 8b; Additional files 1: Figure S2). The xylose subunits interact with the residues along the groove, including residues from the fingers region (Tyr976, Asn44, Glu176, Asn70), palm region (Trp18, Tyr72, Tyr87, Tyr95) and the thumb loop (Pro125, Ser126, Ile127).

Interestingly, the N-terminus lays over the docked xylan hexasaccharide defining a new xylan-binding motif comprising Thr3, Gln4 and Pro5, which we have named the “pinky” motif on account of being at the far end of the fingers region and opposing the thumb loop (Fig. 8b). The presence of the pinky motif in Xyl2 may increase the stability of the binding of the ligand to the enzyme and, therefore, lead to an increase in the rate of xylan degradation. None of the closest structural homologs presents a pinky motif, indicating that this motif may be very specific to a subset of xylanases.

### Kinetic properties

Multiple strategies have been developed for quantifying the activity of GH11 xylanases [45, 46]. Our Xyl2 enzyme assay was based on a specific and rapid colorimetric assay using 4-*O*-methyl-d-glucurono-d-xylan dyed with remazol brilliant blue R (RBB-xylan), a soluble chromogenic substrate [47]. Results are shown in Table 2. The specific activity was determined as 27.2 µmol substrate liberated per minute per mg of enzyme. The *K*_M_ and V_max_ kinetic parameters were obtained by fitting the initial velocity data to a Michaelis–Menten model, with values of *K*_M_ = 40 µM and V_max_ = 2.9 µM/min. The activity was not affected by the presence of the N-terminal hexahistidine tag on the Xyl2 recombinant enzyme (data not shown).

**Table 2:** Kinetic parameters of Xyl2

Activity	Endo-β-(1,4)-xylanase						β-d-xylosidase		
Substrate	RBB-xylan						*p*NP-β-Xyl		
	SA (µmol min^−1^ mg^−1^)^a^	Opt. pH	*K*_M_ (mM)	V_max_ (µM min^−1^)	*k*_cat_ (min^−1^)	*k*_cat_/*K*_M_ (mM^−1^ min^−1^)	SA (µmol min^−1^ mg^−1^)^b^	Opt. pH	*k*_cat_/*K*_M_ (mM^−1^ min^−1^)^c^
Xyl2	27.2	7.0 − 8.0	40 ± 13	2.9 ± 0.6	16.0 ± 1.6	(400 ± 2) × 10^−3^	0.19	6.0	(61 ± 3) × 10^−3^

^a^SA, specific xylanase activity, calculated as μmol Remazol Brilliant Blue R liberated per mg of enzyme per minute at pH 8.0 and 40 °C

^b^ *SA* specific β-d-xylosidase activity, calculated as μmol *p*NP liberated per mg of enzyme per minute at pH 6.0 and 40 °C

^c^The *k*_cat_/*K*_M_ ratio for the β-d-xylosidase activity was calculated directly from a progress curve

Moreover, Xyl2 was active toward *p*NP-β-xylopyranoside (*p*NP-β-Xyl), showing a specific activity of 0.19 µmol substrate liberated per minute per mg of enzyme, measured at 40 °C. Our results demonstrate that Xyl2 can be considered as a potential endo-*β*-1,4-xylanase/*β*-xylosidase enzyme capable of synergically catalyzing those two reactions sequentially. This enzymatic property renders Xyl2 especially interesting as an industrial biocatalyst, owing to the greater economy achievable using a single enzyme to perform two different reactions and to the possibility of tuning the relative catalytic efficiency by adjusting the pH of the reaction medium. Combined, these properties indicate that Xyl2 could be used for the cost-efficient generation of small-sized, fermentable xylooligosaccharides.

**Fig. 9:**
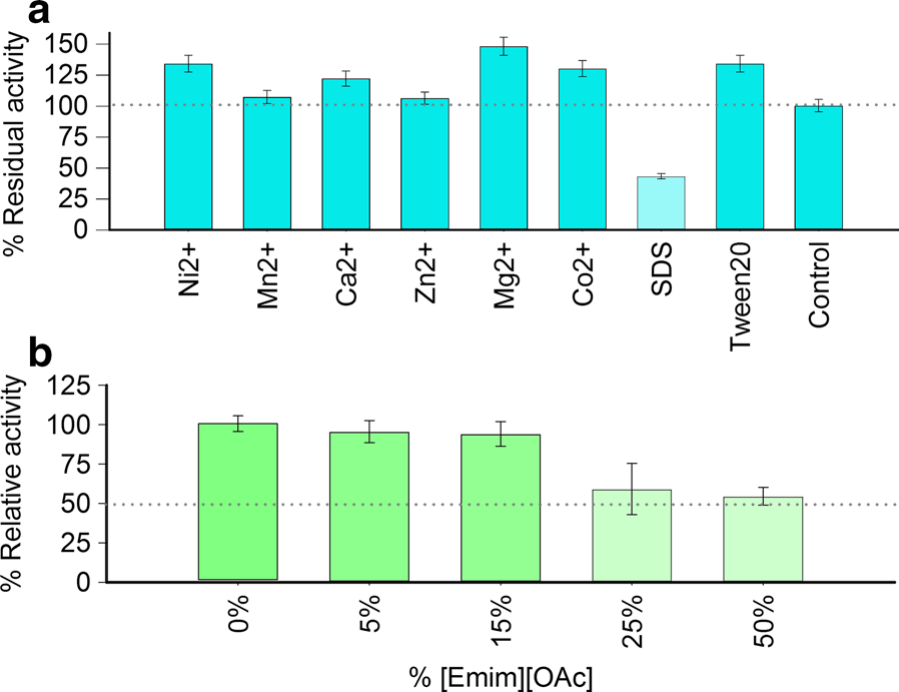
Effect of additives on Xyl2 xylanase activity. **a** Effects of metal cations and ionic and non-ionic detergents. Histogram showing the effect of various metal cations (at 1 mM) and of detergents (0.1 % w/v SDS and 0.05 % w/v Tween-20) as percent increase or decrease of activity with respect to a control reaction under standard conditions (defined in the Experimental section). *Error bars* represent the standard deviation over two replicated experiments. **b** Effect of the [Emim]OAc ionic liquid. Xyl2 was incubated in the presence of variable concentrations of the [Emim]OAc ionic liquid, and residual activity was measured. The activity of the experiment without [Emim]OAc was defined as 100 %

We also tested the effect of various metal cations (Mg^2+^, Ca^2+^, Zn^2+^, Ni^2+^, Co^2+^ and Mn^2+^) on Xyl2 xylanase activity, with the result that most of them appear to activate Xyl2 activity up to 40 %, while in no case is Xyl2 inhibited by the metal concentrations assayed (Fig. 9a). The ranking order of metal ions according to their activation effect is: Mg^2+^ > Ni^2+^ > Co^2+^ > Ca^2+^ > Mn^2+^ and Zn^2+^. The absence of dramatic metal cation effects rules out a strict cofactor requirement for Xyl2. In *Ct*Xyn11, a homologous enzyme (Figs. 6a, b, 2), a Ca^2+^ binding site has been described whose coordination sphere is composed of two conserved residues (Thr10 and Gly13) and a less conserved Asn12 preceded by a histidine residue (PDB 1h1a) [48]. In Xyl2, however, the central His11-Asn12 dipeptide in *Ct*Xyn11 has no clear sequence or structural counterpart in Xyl2, which has Ser10-Gly11, thereby breaking the potential metal coordination sphere. More importantly, the path followed by Xyl2 N-terminal end is markedly different from that in homologous xylanases and, in particular, to that seen in *Ct*Xyn11, which rules out that a similar Ca^2+^ binding site might be present in Xyl2 (Fig. 6c). The tolerance of Xyl2 to inactivation by the assayed metals means that it is largely tolerant to the presence of divalent metal cations in the reaction mixture. Xyl2 is also resistant to moderate concentrations of the ionic detergent sodium dodecyl sulfate (SDS), which elicited an approximately 50 % inhibition at 0.1 % w/v concentration, and the non-ionic polyoxyethylene (20) sorbitan monolaurate (Tween-20) (Fig. 9a).

To investigate the suitability of Xyl2-catalyzed processes in industrial green processes, we also challenged Xyl2 with increasing concentrations of the ionic liquid 1-ethyl-3-methylimidazolium acetate ([Emim]OAc), which is used as a substitute of harsher chemical treatments in environmentally sustainable processes. In our xylanase activity assays, Xyl2 withstood treatment with up to 15 % w/v [Emim]OAc without any reduction in activity and it preserved almost 50 % of residual xylanase activity after being challenged with up to 50 % w/v [Emim]OAc (Fig. 9b). The significant tolerance of Xyl2 to high concentrations of ionic liquids will facilitate the adoption of Xyl2 as a viable biocatalyst in sustainable industrial processes.

### Selection of the optimal support for Xyl2 immobilization

Multiple strategies are under continuous development to promote the use of enzymes as biocatalysts in biofuel and other industrial processes [49]. Among those strategies, enzyme immobilization is considered compulsory for industrial biocatalysts since it typically improves enzyme properties while simultaneously reducing the cost associated with its use, since immobilized biocatalysts can be reused in continuous processes [30]. We sought to enhance the potential industrial applicability of our highly active preparation of *F. oxysporum* Xyl2 by immobilization through approaches based on covalent attachment to solid supports and crosslinking. To maximize the amount of activity retained in the Xyl2-immobilized support, we optimized crucial parameters including reaction time, enzyme load, pH, temperature, buffer composition, protein-stabilizing additives and support features, all of which can be tuned to improve enzymatic properties [30, 50].

Since the nature of the support greatly influences enzyme-immobilized activity through conformation-controlled effects, we decided to screen multiple supports and immobilization strategies [51]. A summary of the immobilization results for Xyl2 is shown in Table 3. In summary, Xyl2 was efficiently immobilized on different supports and through various non-covalent and covalent binding modes to functionalized agarose beads or cross-linked glutaraldehyde–chitosan. Although Xyl2 was able to bind to every support matrix tested, remarkable differences were observed with respect to residual activity. As shown in Table 3 and Additional Fig. 3b in Additional file 1, best results (most active immobilized enzyme) were achieved when Xyl2 was covalently bound to low functionalized agarose matrices according to the GL-1 > GL-3 > AM-1 ranking order. Indeed, in those lower functionalized supports, immobilized Xyl2 activity was enhanced over that of the free enzyme (Table 3). This effect may be attributable to the development of an optimal microenvironment in the solid support, the adoption of an optimal protein orientation and/or to an increase in conformational flexibility, among other factors [30]. The poorer activity observed for Xyl2 immobilized on highly functionalized agarose or on nickel-affinity resin (Table 3) correlates with higher immobilization mass yields, which might explain the lower activity through greater steric hindrance effects. Other explanations are possible to account for the reduced activity of biocatalysts immobilized on glutaraldehyde–chitosan or some agarose supports, including inhibitory conformational changes and loss of flexibility as a consequence of the immobilization procedure [50]. Despite its simplicity and economy, cross-linked enzyme aggregates (CLEAs) are subject to inactivating aggregation caused by the immobilization method, which typically results in the reduction of enzyme activity owing to substrate mass transfer limitations [52, 53]. We also selected supports and immobilization methods that could maximize other desirable enzyme properties other than activity, such as thermal stability, since stabilization by multicovalent point attachment is a well-established procedure to accomplish both goals [54, 55].

**Table 3:** Immobilization yield (IY) and residual activity (RA) in the immobilization of Xyl2 by different approaches

Immobilization method	RA (%)	IY (%)
Free enzyme	100.0	−
CLEA (25 % glutaraldehyde)	8.5	*65*
His-select affinity resin	4.2	>*95*
Glutaraldehyde–chitosan	ND	35
AM-1	*133.0*	25
AM-2	46.0	25
AM-3	*58.0*	27
AM-4	48.0	25
GL-1	*157.8*	32
GL-2	39.6	*61*
GL-3	*151.3*	33
GL-4	34.0	*68*
GL-5	18.2	*76*

*ND* not detected. *IY/RA* values above 50 % are shown in italic face

### Temperature and pH effects on free and immobilized Xyl2

Agarose beads have long been recognized as suitable supports for enzyme immobilization, resulting in highly stable enzyme preparations across an extremely wide range of conditions [56]. The higher stability in covalent multipoint binding supports is thought to be associated with a reduction in conformational flexibility and thermal vibrations of the immobilized enzyme compared with the free form in solution [57, 58].

**Fig. 10:**
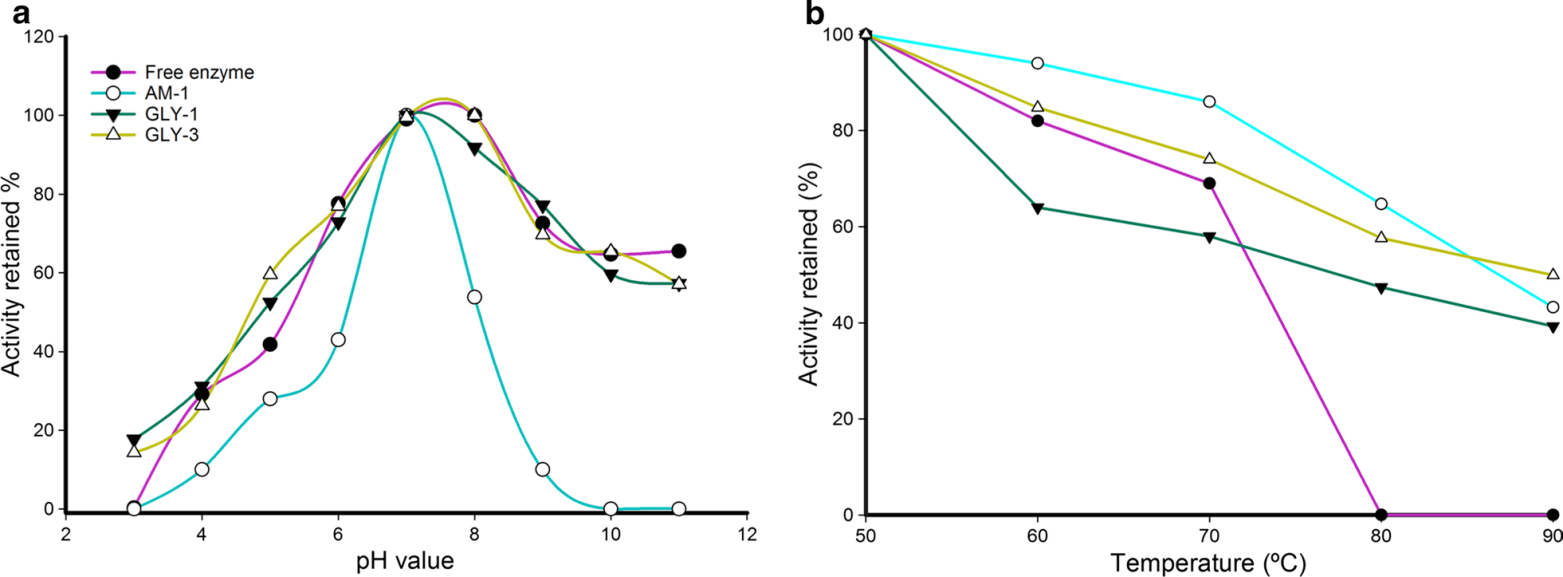
pH and temperature stability of free and immobilized Xyl2. **a** pH stability of free and different immobilized complexes of Xyl2. Enzyme activity was measured at 40 °C in a pH range between 3 and 11 and expressed as percent residual activity. **b** Thermostability of free and immobilized Xyl2 over different supports. Activity was measured after incubation at different temperatures for 30 min, and expressed as percent residual activity

To further investigate the kinetic and stability properties of immobilized Xyl2, we tested the effect of pH and temperature on its endo-β-1,4-xylanase activity. The xylanase activity was measured in a pH range between 3 and 11, and the results were normalized as percent residual activity with respect to the pH of highest activity, to which 100 % was assigned (Fig. 10a). The results of the pH scouting on free and immobilized Xyl2 demonstrated that the activity profile of both enzyme forms peaks at pH 7–8 and is, thus, largely independent of the free or immobilized state of the enzyme, at least for the glyoxal-based GL-1 and GL-3 supports. For Xyl2 immobilized on AM-1 resin, the position of the pH optimum is not altered, but the width of the pH profile becomes narrower around pH 7.

To ascertain the temperature dependence of the activity profile of Xyl2, the enzyme was first incubated at temperatures between 50 and 90 °C for 30 min, and then the activity was measured and expressed as percent residual activity with respect to the activity value measured at 50 °C, which was assigned 100 %. As shown in Fig. 10b, immobilization of Xyl2 brought about a steady shift of the thermal stability profile toward more elevated temperatures compared to the free enzyme. For all tested supports, immobilized Xyl2 became thermostable up to at least 80 °C, with ~50 % of residual activity at 90 °C. The above thermal stability profiles (Fig. 10b) were further used to calculate the kinetics of thermal inactivation of Xyl2, which was summarized as T50 % values, i.e., the temperature at which 50 % of residual activity remains [59]. While T50 % for free Xyl2 was 72 °C, the T50 % for GL-1-immobilized Xyl2 increased to 77 °C. More remarkable was the greater thermostability shift that was attained after immobilizing Xyl2 over AM-1 and GLY-3 supports, with T50 % of 86 and 90 °C, respectively. The difference in thermostability between free and GL-1-immoblilized Xyl2 and multipoint immobilized Xyl2 (AM-1/GLY-3) amounted to as much as 18 °C. These T50 % values compare well with the optimal temperatures measured for thermophilic xylanases; among them are the enzymes from bacteria such as *Dictyoglomus thermophilum* XynB (optimal temperature 75 °C) [60], *Thermobifida fusca* NTU22 Xyl11 (70 °C) [61], and *Thermopolyspora flexuosa* XynA (80 °C) [62], and fungal xylanases from *Neocallimastix patriciarum* XynCDBFV (optimal temperature 65 °C) [63] and *Chaetomium thermophilum* Xyn11A (optimal temperature 80 °C) [48]. Therefore, these comparisons qualify Xyl2 and, especially, AM-1 and GLY-3 immobilized Xyl2, as thermophilic/thermostable enzyme preparations.

### Suitability of Xyl2 as a biocatalyst in industrial processes

The success of many industrial bioprocesses that involve xylanases and other glycosyl hydrolases hinges on an appropriate selection of the biocatalyst in terms of activity toward the specific substrates, stability under demanding reaction conditions, and versatility. The latter property ensures that the biocatalyst can be adapted as required when the bioprocess is optimized or modified. In this context, Xyl2 offers several advantageous features as a promising biocatalyst in comparison with other commercially available xylanases from the same or alternative sources. The specific activity of Xyl2 (27.2 U/mg) is comparable, even without sequence or process optimization, with those of well-studied xylanases such as Xys1 from *Streptomyces halstedii* JMO (32 U/mg) [64] and several commercial thermostable xylanases (Sigma-Aldrich) (40 U/mg) [65]). It should be noted that a direct comparison of xylanase activity values might be regarded as only approximate owing to the diversity of experimental conditions and measurement protocols in use [45]. Although some xylanases from *F. oxysporum* [23] have been reported to have higher activity than Xyl2, they are not stable at temperatures up to 45 °C, where they lose more than 50 % of the initial activity after 60 min incubation, thereby rendering their use impractical in many industrial processes [25]. In contrast, the greater thermotolerance of Xyl2 in solution (~70 % of residual activity after 30 min of incubation at 70 °C and T50 % for free Xyl2 of 72 °C) and, especially, after immobilization on a variety of solid supports (T50 % of 77, 86 and 90 °C, for GL-1, AM-1 and GLY-3 supports, respectively), increases the potential industrial applicability of Xyl2.

The robustness of Xyl2 activity in the presence of ionic liquids is a prerequisite for its application to processes where ILs are employed for the pretreatment of lignocellulosic biomass. Given the high costs associated with the handling and disposal of the acidic/basic and organic solvents used in traditional biomass pretreatments, and the higher energy consumption and worse environmental impacts associated with them, the use of ILs in modern, greener alternative processes is bound to increase over time. The suitability of Xyl2 as an efficient biocatalyst in the presence of up to 15 % w/v [Emim]OAc, without effect on xylanase activity, is, therefore, an important property to reckon.

Furthermore, another unique property of Xyl2 is its activity toward *p*NP-β-Xyl (0.19 U/mg), which, to the best of our knowledge, has not been reported in any of the GH11 xylanases previously described in this fungus. This β-xylosidase activity exhibited by Xyl2 would be advantageous for the development of greener industrial processes (e.g., biofuel and nutrition) by combining it with the endo-β-1,4-xylanase activity to create enzyme preparations harboring two essential hydrolytic activities rather than only one. These one-enzyme preparations would save cost and help simplify process design by reducing the need to produce, store, and deliver two different enzymes when Xyl2 could deliver both endo-β-1,4-xylanase and β-xylosidase activities.

Finally, an increase in enzyme activity (157.8 % of residual activity when Xyl2 was immobilized in glyoxal support GL-1) and thermostability (~50 % of residual activity after 30 min of incubation at 90 °C) was observed when Xyl2 was immobilized through amine coupling on GL supports. This phenomenon, which has important practical consequences for the continuous use and recycling of biocatalysts, has not been reported previously for other xylanases from non-thermophilic organisms immobilized onto glyoxyl agaroses (compare residual activity of 66.8 % on lab-activated agarose beads versus 157.8 % on GL-1) [66].

Therefore, it can be concluded that Xyl2 exhibits promising enzymatic and stability properties for the biodegradation of xylan under alkaline conditions at high temperature, which are further improved by immobilization on suitable supports. All these properties combined, render Xyl2 a promising candidate enzyme for its development as an industrially viable biocatalyst.

## Conclusions

We have identified *F. oxysporum* Xyl2 as a promising GH11 endo-β-1,4-xylanase and cloned a synthetic gene encoding an N-terminally hexahistidine-tagged version of Xyl2 for recombinant expression of the enzyme, structural studies and functional characterization. The crystal structure of Xyl2 at 1.56 Å has revealed an atomic-level view of the active-site groove from which the catalytic mechanism can be inferred. A second crystal structure obtained with a single β-xylopyranoside unit bound highlights position-2 as the preferred anchoring place for xylan chains, and provides a substrate-binding site for Xyl2 residual β-xylosidase activity. Kinetic and stability studies of Xyl2 in both free form and as an immobilized enzyme in a variety of solid supports demonstrate the potential for this highly active enzyme to act as an efficient xylanase/β-xylopyranoside biocatalyst in a variety of challenging conditions, as those demanded by industrial applications.

## Methods

### Materials and reagents

General chemical compounds, 1-ethyl-3-methylimidazolium acetate ([Emim]OAc), chitosan, glutaraldehyde and 4-*O*-methyl-d-glucurono-d-xylan dyed with Remazol brilliant blue R (RBB-xylan) were from Sigma-Aldrich. *p*NP-β-d-xylanopyranoside (*p*NP-Xyl) was purchased from Merck-Millipore. The HisTrap column for protein purification was from GE Healthcare. Agarose resins for protein immobilization were acquired from ABT (glyoxal and aminoethyl supports) and Sigma-Aldrich (Ni-agarose support).

### Recombinant Xyl2 production

An expression plasmid derived from pETM-11 was constructed using an optimized synthetic gene sequence (Genscript) encoding *F. oxysporum* Xyl2 sequence (UniProt entry X0M5X0_FUSOX). Recombinant full-length Xyl2, except for the predicted peptide leader sequence, spanning amino acids 19–232, was expressed in *E. coli* BL21(DE3) cells as an N-terminal hexahistidine-tagged enzyme. An overnight starter culture (1 ml) supplemented with 25 µg/ml kanamycin was used to inoculate 0.5 l Power Broth (AthenaES), and the cells were allowed to grow at 37 °C until the culture reached an OD_600_ 0.8, time at which the temperature was reduced to 20 °C and Xyl2 expression was induced by addition of 0.5 mM IPTG. The cells were collected 18 h later by centrifugation, and the cell pellet was resuspended in buffer A (50 mM Tris–HCl pH 8.0, 0.5 M NaCl and 20 mM imidazole) supplied with one protease inhibitor cocktail (Roche) lozenge and 1 mM PMSF. The cell suspension was then lysed by sonication (70 % output power, 10 min) and cell debris removed by centrifugation at 12,500 rpm for 30 min at 4 °C. The resultant supernatant was clarified by filtration through a 0.45-µm filter membrane and loaded onto a 5-ml HisTrap column previously equilibrated with buffer A. Next, the column was thoroughly washed with 10 column volumes of buffer A, 10 column volumes of buffer B (buffer A with 50 mM imidazole), and the bound Xyl2 was finally eluted with buffer C (buffer A with 250 mM imidazole). Elution fractions containing pure Xyl2 were pooled together, concentrated and buffer-exchanged into storage buffer (10 mM Tris–HCl pH 7.6) before snap-freezing them in liquid nitrogen. Roughly half of the eluted Xyl2 was treated with rTEV protease to cleave off the hexahistidine tag. Cleavage was performed in dialysis against buffer A overnight at 4 °C. Next morning, the digestion was loaded onto a fresh HisTrap column, and cleaved Xyl2 was collected in the flow-through, concentrated and buffer-exchanged into storage buffer. Both histidine-tagged and tag-cleaved Xyl2 protein preparations were tested immediately after purification for xylanase activity by spotting 5 µl enzyme onto Luria–Bertani agar plates containing 4 mM Remazol brilliant blue R-d-xylan (RBB-xylan) and incubated at 40 °C for 3–4 h. Xylanase activity gave rise to a white depletion halo around the spotted enzyme.

### Crystallization, structure determination and refinement

Before attempting crystallization of Xyl2, a commercial pre-crystallization test (Hampton Research) was used to select a suitable protein concentration for more extensive crystallization screenings, which was finally set to 5 mg/ml. The full JCSG-plus sparse matrix and the PACT premier systematic PEG/Ion/pH screenings (Molecular Dimensions) were performed by the sitting-drop vapor-diffusion method using drops containing 1 μl Xyl2 and 1 μl crystallization condition at 20 °C. Small prism-like crystals (100 μm in their longest dimension) appeared within 1 week under several conditions containing 0.1 M sodium citrate pH 5.0 and varying ammonium sulfate concentrations. To obtain structural information on Xyl2 ligand complex, crystals were soaked with 10 mM methyl-β-d-xylopyranoside (MBX) for 10–30 min. Both unliganded and Xyl2/MBX crystals were then cryoprotected with CryoMix #1 (Molecular Dimensions), mounted in standard MicroMount (MiTeGen) and flash-frozen in liquid nitrogen.

Complete X-ray diffraction data sets at a wavelength of 0.98002 Å were collected from unliganded Xyl2 crystals at the PROXIMA 2A beamline (Synchrotron Soleil, Paris, France) at 1.56 Å resolution and from crystals of Xyl2 in complex with MBX at the BL13-XALOC beamline (ALBA Synchrotron, Barcelona, Spain) at 2.84 Å resolution. Data sets were integrated with XDS [67] and scaled with Aimless [68] from the CCP4 suite of programs (Table 1) [69].

The structures of unliganded and liganded Xyl2 were determined by the molecular replacement method using the program PHASER [70] from the PHENIX program suite [71] and built, refined and validated using *Coot* [72], phenix.refine [73] and MolProbity [74]. The crystal structure of an unliganded mutant endo-β-1,4-xylanase II from *Trichoderma reesei* (PDB 4hkl) [40] was used as search model for the unliganded Xyl2 after modifying the coordinates with *CHAINSAW* [75] according to a sequence alignment (64.7 % identity). Next, the structure of Xyl2/MBX was determined using the refined unliganded Xyl2 structure as model.

All figures showing protein structures were rendered with PyMOL [76].

### Docking

The β-1,4-xylopyranoside hexasaccharide (XYP6) was docked into the Xyl2 active site with AutoDock Vina [43, 44]. Before docking, the crystal structure of *T. reesei* Xyn11 with a bound XYP6 ligand (PDB 4hk8) [40] was superimposed on that of the Xyl2/MBX complex, and the superimposed coordinates of Xyl2 and XYP6 were used as the starting point for the docking runs. The grid scale for docking was set as 60 × 90 × 60 Å based on a grid module with 0.375 Å spacing between the grid points. Given the functionally relevant changes observed at basic and acidic pH between the side chain conformations of the catalytic residues, we ran docking experiments at pH 5 and 7. We modeled both pH extremes by suitably adding hydrogen atoms and assigning atomic charges with pdb2pqr [77] and Propka [78, 79]. Gasteiger charges [80] were assigned to both the protein and the ligand only at pH 7.5 (with a total charge of 6), while at pH 5, the charges calculated with Propka were preserved for docking (total charge of 9). A hundred independent docking runs were performed. During the calculation, we kept all the ten torsional bonds of the ligand free. In contrast, Xyl2 was kept rigid; at pH 5, the liganded Xyl2 structure was used, while at pH 7.5, the side chain conformations of Tyr72 and Glu176 were grafted from PDB code 4hk8. At the end, the bioactive pose was selected as the conformation with the most cluster members and the lowest Xyl2/XYP6 interaction energy.

### Xylanase enzyme assays

The Xyl2 xylanase enzyme assay was based on a specific and rapid colorimetric assay using 4-*O*-methyl-d-glucurono-d-xylan dyed with Remazol brilliant blue R (RBB-xylan), a soluble chromogenic substrate [47]. For the free enzyme (His-Xyl2 or Xyl2 fractions, with or without the N-terminal hexahistidine tag, respectively), single-point activity measurements were determined by adding 25 µl 3.2 mM substrate dissolved in 50 mM Tris–HCl pH 8.0 to 46 µg of total enzyme and incubating the reaction mixture at 40 °C for 10 min. The reaction was stopped by addition of 150 µl 96 % ethanol under vigorous mixing. After 15 min of incubation, the reaction was thoroughly mixed and centrifuged at maximum speed for 1 min. The amount of soluble enzyme-released RBB-dyed product was determined by measuring the absorbance at 590 nm, and specific activity was calculated by interpolating into a standard curve as μmol RBB-xylan liberated per mg of protein and per minute at pH 8.0 and 40 °C. To determine the kinetic parameters, the RBB-xylan concentration was varied from 0 to 55 mM and each initial velocity calculated by averaging three independent measurements. The *K*_M_ and V_max_ values were calculated by fitting the initial velocity data to a Michaelis–Menten kinetic model using the SigmaPlot software v. 13 [81].

For immobilized enzyme preparations, 200 µl of 3.2 mM substrate dissolved in 50 mM Tris–HCl pH 8.0 was added to each immobilized enzyme preparation. The mixture was incubated for 120 min at 40 °C with continuous agitation, and the reaction was stopped by adding 500 µl 96 % ethanol. Further steps were performed as previously described for the free enzyme.

Complementary β-xylosidase activity was tested with a colorimetric assay based on the accumulation of *p*NP released by enzyme action on the artificial substrate *p*NP-β-xylopyranosyde. Continuous spectrophotometric detection was performed in an Eppendorf spectrophotometer (BioSpectrometer) with temperature controlled at 40 °C. The reaction was started by adding 0.7 µg of Xyl2 to an assay mixture containing 5 mM substrate solution in 50 mM sodium phosphate buffer pH 6.0. A linear increase in absorbance at 405 nm was followed during 5 min. One unit of β-xylosidase activity was defined as μmol *p*NP liberated per mg of enzyme per minute at pH 6.0 and 40 °C.

The effect of various reagents on the xylanase activity of Xyl2 was tested at the standard reaction conditions by adding them to a specified final concentration: 1 mM for divalent metal cations (Mg^2+^, Ca^2+^, Zn^2+^, Ni^2+^, Co^2+^ and Mn^2+^), 0.05 % w/v polyoxyethylene (20) sorbitan monolaurate (Tween-20), 0.1 % w/v sodium dodecyl sulfate (SDS), and 0–50 % w/v ionic liquid 1-ethyl-3-methylimidazolium acetate ([Emim]OAc).

### Protein immobilization

Xyl2 was immobilized on different supports by a variety of approaches, including covalent coupling and crosslinking (schematized in Additional Fig. 3a in Additional file 1). Immobilization steps were performed at room temperature or at 4 °C, and supernatants and washes were recovered after each step to determine the percentage of non-immobilized protein. Supports employed were commercially available or synthetized and activated in this work. The following equations were used, where C_0_ and C_F_ are the enzyme concentrations at starting and final immobilization times and A_0_ and A_F_ are the free and immobilized enzyme activity:

*Immobilization yield* $$({\text{IY}}\,\% ) = 100 \times \frac{{C_{0}\, {-}\, C_{\text{F}} }}{{C_{0} }}$$

*Residual activity* $$({\text{RA}}\,\% ) = 100 \times \frac{{A_{0} \,{-} \,A_{\text{F}} }}{{A_{0} }}$$

#### His-select agarose beads support

Hexahistidine-tagged His-Xyl2 was non-covalently immobilized onto His-Select nickel agarose beads (Sigma-Aldrich). For that purpose, 50 µl of His-Select resin was twice washed with double-distilled water and equilibrated with a buffer containing 10 mM Tris–HCl buffer pH 7.6. Next, 0.75 mg His–Xyl2 was loaded onto the resin, and the total volume was brought to 250 µl with equilibration buffer. The suspension was incubated on a rotary shaker for 3 h at room temperature. Immobilized His–Xyl2 was obtained by centrifugation, and the supernatant was used to quantify the remaining unbound enzyme.

#### Glutaraldehyde–chitosan support

To prepare the glutaraldehyde–chitosan support (GA–chitosan) for immobilization, chitosan powder (0.5 g) was dissolved in 50 ml 0.1 M HCl, and 2.5 % (v/v*)* glutaraldehyde was added to the mixture. After incubating the suspension at 30 °C for 3 h with vigorous agitation, 1 ml 1 M NaOH was added under continuous agitation to precipitate the support. The solution was centrifuged at 4000 rpm for 10 min to retrieve the support, which was then gently washed with double-distilled water and equilibrated with a buffer containing 50 mM Tris–HCl pH 7.6. The GA–chitosan was dried and stored in 20 % ethanol at 4 °C until use.

Fifty (50) mg of GA–chitosan was washed twice with double-distilled water and equilibrated with 10 mM Tris–HCl buffer pH 7.6 for immobilization. Xyl2 (0.5 mg) was mixed with the GA–chitosan support and kept at 4 °C for 24 h with continuous rotational agitation to allow protein covalent immobilization. At the end of the incubation, samples were centrifuged and remaining unbound enzyme was quantified in the supernatant and wash fractions.

#### Crosslinking with glutaraldehyde

To prepare cross-linked aggregates, 1 mg Xyl2 was first precipitated by slow addition of ammonium sulfate to 80 % (w/v) final concentration, with constant stirring for 24 h at 4 °C. Crosslinking was then performed by dropwise addition of glutaraldehyde to 2 % (v/v) final concentration over 3 h at room temperature. The resulting CLEAs were isolated by centrifugation and washed with 50 mM Tris–HCl pH 7.6 and double-distilled water.

#### Affinity coupling on functionalized agarose supports

Xyl2 was immobilized on two classes of agarose supports, aminoethyl and glyoxal agarose beads, following manufacturer’s instructions. The two types of agarose supports differed in binding capacity (very low, low, high and very high activation level) and degree of matrix crosslinking (4 and 6 %) (Additional file 1: Table 1). This is the first report where a xylanase is immobilized on aminoethyl agarose beads, whereas previous reports on immobilized agaroses dealt with glyoxal agarose supports [66, 82, 83].

Aminoethyl agarose (AM) resins allow protein immobilization to occur through covalent coupling via the carboxylate moieties of solvent-exposed aspartates, glutamates and C-termini. For these supports (AM-1, AM-2, AM-3 and AM-4), 10 µl of each aminoethyl resin was rinsed with double-distilled water and equilibrated with 10 mM Tris–HCl pH 7.6. Different amounts of Xyl2 were mixed with the pre-equilibrated resins according to their binding capacity, and adding EDC to 60 mM final concentration started the covalent binding reaction that was gently stirred with orbital shaking at 4 °C. To stop the reaction, the resins were centrifuged (13,500 rpm, 2 min), rinsed with double-distilled water followed by 1 M NaCl, and then resuspended in double-distilled water. The supernatant and washes were saved for analysis.

For the glyoxal agarose resins (GL-1 to GL-5), Xyl2 was covalently attached through exposed amine groups (solvent exposed lysine side chains and the N-terminus). Ten (10) µl of each resin was washed, equilibrated with 0.1 M sodium bicarbonate pH 7.0 and mixed with varying amounts of Xyl2 depending on the nominal binding capacity of each resin. Binding was allowed to proceed at 4 °C with constant orbital shaking. Adding 0.1 mg sodium borohydride and incubating for 30 min with shaking stopped the reaction. Last, the resins were washed with 10 mM Tris–HCl pH 7.6, keeping the supernatant and washes for analysis.

### pH optimum

The influence of pH on the activity of the free and immobilized Xyl2 was investigated with the standard RBB-xylan xylanase activity assay. Several buffers were tested at 50 mM in a wide pH range between 3 and 11: sodium acetate pH 3.0–5.0, sodium phosphate pH 6.0 and 7.0, and Tris–HCl for the 8.0–11.0 interval at 1 pH unit steps.

### Thermal stability

The effect of temperature on enzyme stability was assayed over a temperature range spanning 50–90 °C in 10 °C steps. Free and immobilized Xyl2 were incubated at each temperature for 30 min, and then, the remaining xylanase activity was assayed as described above. The percent residual activity at each given temperature with respect to the activity measured at 50 °C (set to 100 %) was used as a quantitative measure of thermal stability.

## Additional file

10.1186/s13068-016-0605-z Additional Fig. 1. Schematic representation of Xyl2 topology. Additional Fig. 2. Docking of a xylose hexaoligosaccharide on Xyl2 (pH 5). Additional Fig. 3. Schematic representation of the rationale for random enzyme immobilization via the carrier or carrier-free approaches. Negative correlation between Xyl2 activity yield and functionalization degree in high and low agarose supports. Additional Table 1. Guiding values for binding capacities of commercial agarose beads employed for Xyl2 immobilization.

## Abbreviations

GH11: family 11 glycoside hydrolase
GH10: family 10 glycoside hydrolase
ILs: ionic liquids
[Emim]OAc: 1-ethyl-3-methylimidazolium acetate
TEV: tobacco etch virus
CBB: coomassie brilliant blue
MBX: methyl-β-d-xylopyranose
CBM: carbohydrate-binding module
RBB-xylan: 4-*O*-methyl-d-glucurono-d-xylan dyed with remazol brilliant blue R
*p*NP-β-Xyl: *p*NP-β-xylopyranoside
IPTG: isopropyl-β-d-thiogalactopyranoside
SDS: sodium dodecyl sulfate
SDS-PAGE: SDS-polyacrylamide gel electrophoresis
PMSF: phenylmethanesulfonyl fluoride
CLEAs: cross-linked enzyme aggregates
XYP6: β-1,4-xylopyranoside hexasaccharide
GA–chitosan: glutaraldehyde–chitosan support
AM: aminoethyl agarose
GL: glyoxal agarose resins
IY: immobilization yield
RA: residual activity
