# Supplementary material for: Structural and functional characterization of a highly stable endo-β-1,4-xylanase from Fusarium oxysporum and its development as an efficient immobilized biocatalyst
Source: Biotechnol Biofuels. 2016 Sep 5;9:191. doi: 10.1186/s13068-016-0605-z (PMC5011838; doi:10.1186/s13068-016-0605-z)
Supplement: Supplementary file 1 — 10.1186/s13068-016-0605-z Additional Fig. 1. Schematic representation of Xyl2 topology. Additional Fig. 2. Docking of a xylose hexaoligosaccharide on Xyl2 (pH 5). Additional Fig. 3. Schematic representation of the rationale for random enzyme immobilization via the carrier or carrier-free approaches. Negative correlation between Xyl2 activity yield and functionalization degree in high and low agarose supports. Additional Table 1. Guiding values for binding capacities of commercial agarose beads employed for Xyl2 immobilization. [file 13068_2016_605_MOESM1_ESM.pdf]

Additional Information

Structural and Functional Characterization of a Highly Stable Endo-β-1-4-xylanase from *Fusarium oxysporum* and Its Development as an Efficient Immobilized Biocatalyst

Sara Gómez[1], Asia M. Payne[1], Martin Savko[2], Gavin C. Fox[2], William E. Shepard[2], Francisco. J. Fernandez[1,3] and M. Cristina Vega[1]*

* Correspondence: cvega@cib.csic.es.

---

[1] Structural and Quantitative Biology Department, Center for Biological Research (CIB-CSIC), Madrid, Spain. E-mail: cvega@cib.csic.es

[2] Synchrotron SOLEIL, Gif-sur-Yvette, France.

[3] Abvance SRL, Madrid, Spain.

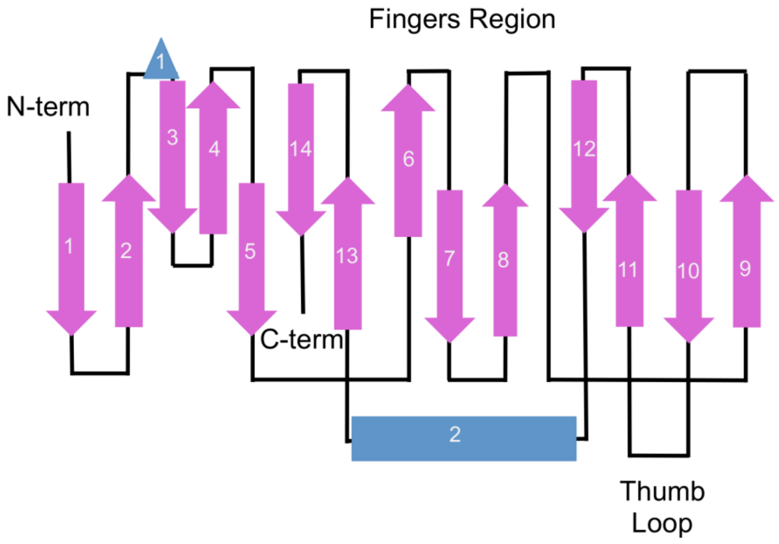

Additional Fig. 1. Schematic representation of Xyl2 topology. Strands are represented as arrow-headed rectangles, in pink, the α-helix is in blue, the 3_{10}A helix in cyan, and all connections are drawn as black lines.

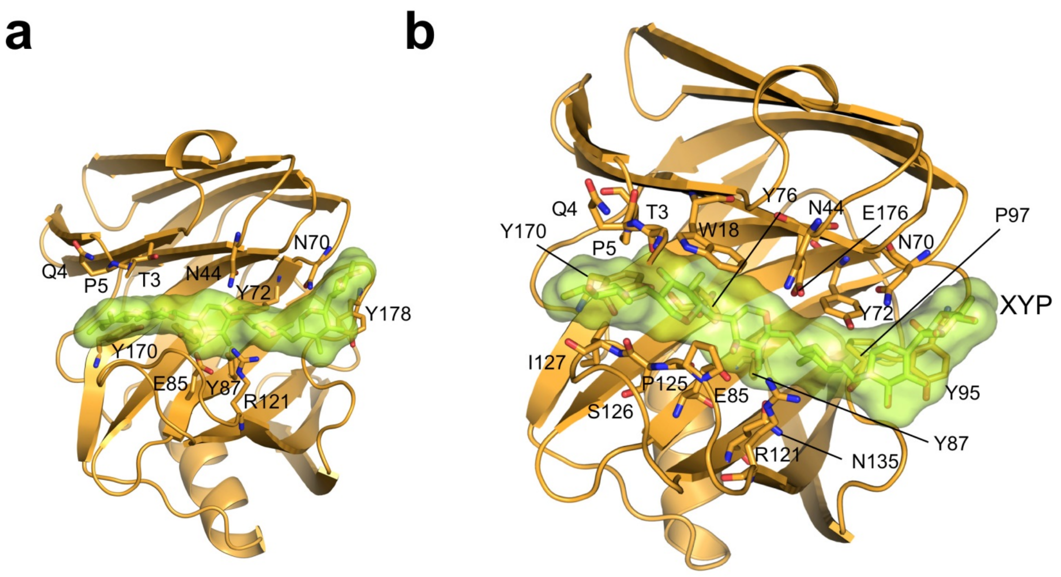

Additional Fig. 2. Docking of a xylose hexaoligosaccharide on Xyl2 (pH 5). This figure complements the docking results shown in Fig. 7 of the main text. The Xyl2 structure, represented as cartoon in orange, is that of the Xyl2/MBX crystal structure at 2.84 Å including the experimentally determined rotameric conformations of Tyr72 and Glu176 side chains. The hexasaccharide can be equally docked without loss of free energy or unfavorable interactions, despite the “flipped over” rotamers of the catalytic residues. Most of the interactions are established with the deepest bound section of the xylan chains (specifically, xylose subunit -2).

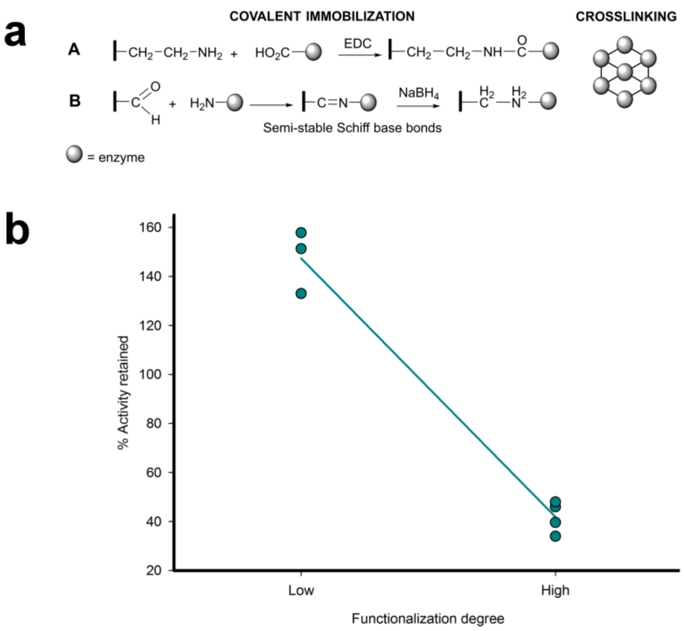

Additional Fig. 3. **(a)** Schematic representation of rationale for random enzyme immobilization via the carrier or carrier-free approaches. Covalent binding (A, aminoethyl supports; B, glyoxal supports) and crosslinked enzyme aggregates (CLEAs). **(b)** Negative correlation between Xyl2 activity yield and functionalization degree in high and low agarose supports. For visualization, supports with very high and very low functionalization degrees were discarded. The supports with low (AM-1, GL-1 and GL-3) or high (AM-2, AM-4, GL-2 and GL-4) functionalization degrees are depicted on two separate vertical lines intersecting an arbitrary horizontal coordinate, with the vertical axis representing the percent activity yield or residual activity. The negative correlation was calculated by linear regression (R^2 = 0.9768).

Additional Table 1. Guiding values for binding capacities of commercial agarose beads employed for Xyl2 immobilization.

**Support**	**Matrix**[1]	**Functionalization degree**[2]	**Binding capacity**[3]
Ni-Agarose resin	6%	6-18	≥15
Aminoethyl Low (AM-1)	6%	15-25	~14
Aminoethyl High (AM-2)	6%	40-60	~30
Aminoethyl Very low (AM-3)	4%	3-6	~5
Aminoethyl High (AM-4)	4%	40-60	~30
Glyoxal Low (GL-1)	4%	15-25	~10
Glyoxal High (GL-2)	4%	40-60	~20
Glyoxal Low (GL-3)	6%	15-25	~10
Glyoxal High (GL-4)	6%	40-60	~20
Glyoxal Very High (GL-5)	6%	80-100	~30

[1] Agarose %; [2] μmol Ni^{2+}, aminoethyl or glyoxyl /ml gel; [3] mg protein/ml gel.
